# Vaccination Coverage Among Persons with Asthma — United States, 2010–2011 Influenza Season

**Published:** 2013-12-06

**Authors:** Michael E. King, Peng-jun Lu, Alissa O’Halloran, Helen Ding, Matthew J. Lozier

**Affiliations:** Div of Environmental Hazards and Health Effects, National Center for Environmental Health; Immunization Svcs Div, National Center for Immunization and Respiratory Diseases; EIS Officer, CDC

Asthma was the most common underlying condition among persons hospitalized with pandemic influenza A (H1N1) virus infection in 2009 (1). Although persons with asthma are not more likely than others to get influenza, influenza can make asthma symptoms worse, trigger asthma attacks, and lead to pneumonia or other complications that result in hospitalization and even death.* During 1964–2010, the Advisory Committee on Immunization Practices (ACIP) recommended that all adults and children aged ≥6 months with asthma receive an influenza vaccination annually (2). Beginning with the 2010–11 influenza season, ACIP expanded its annual vaccination recommendation to include all persons aged ≥6 months, while emphasizing that protection of persons at higher risk for influenza-related complications continue as a focus of vaccination efforts (2). To provide the first update of national vaccination coverage among persons aged ≥2 years with asthma since the new ACIP recommendation, CDC analyzed data from the 2010 and 2011 National Health Interview Survey (NHIS). This report describes the results of that analysis, which indicated that influenza vaccination during the 2010–11 season among persons with asthma was 50%, up from 36% 5 years earlier (3). However, vaccination coverage across all age groups, including among those with health insurance, a usual place for health care, and one or more health-care visits in the past 12 months, remained well below *Healthy People 2020* targets† of 80% for children aged 6 months–17 years and 90% for adults aged ≥18 years who are at high risk. These findings highlight the need to educate health-care providers and persons with asthma about the importance of annual influenza vaccination.

NHIS is an annual, in-person survey of the noninstitutionalized U.S. civilian population. It is based on a multistage sampling of households (4). From each family surveyed, one sample child (if present) and one sample adult are randomly selected, and information about receipt of influenza vaccination in the previous 12 months is collected. This analysis used 2010 and 2011 NHIS data to estimate influenza vaccination coverage among persons with current asthma§ aged ≥2 years¶ during the 2010–11 influenza season. To better assess influenza vaccination coverage for the 2010–11 season, data from respondents interviewed during September–June and vaccinated during August–May were analyzed. For missing vaccination month and year, information was imputed from donor pools matched for week of interview, age group, region of residence, and race/ethnicity. The Kaplan-Meier survival analysis procedure was used.**

All analyses were conducted using statistical software to account for the complex sample design. Vaccination status was stratified by characteristics known to be associated with influenza vaccination, including age group, race/ethnicity, family income relative to family size, health insurance coverage, number of health-care visits in the past year, and having a usual place for health care (3,5,6). Weighted estimates of vaccination coverage were compared using t-tests, with statistical significance defined as p<0.05.

The response rates for the 2010 and 2011 NHISs were 79.5 and 82.0%, respectively. Responses from 32,636 persons aged ≥2 years were analyzed. Of those, 2,809 (8.6%) reported having (or were reported to have) current asthma. Vaccination coverage for the 2010–11 season among persons with current asthma was 49.6%, compared with 37.5% among those without current asthma (p<0.05) (Table 1). Among persons with current asthma, those aged 50–64 years and ≥65 years had the highest vaccination coverage (61.7% and 76.5%, respectively). For all age groups, a higher proportion of persons with current asthma received influenza vaccination than did those without current asthma (p<0.05) (Table 1). Vaccination coverage among persons with asthma who experienced an asthma attack in the preceding 12 months did not differ significantly from the coverage of persons with asthma who did not have an asthma attack or an emergency department (ED)/urgent care visit in the preceding 12 months. Vaccination coverage was also similar among persons with asthma who had an ED/urgent care visit in the preceding 12 months to the coverage of persons with asthma who did not have an asthma attack or ED/urgent care visit in the preceding 12 months.

For all persons, vaccination coverage increased as the number of health-care visits over the past year increased, and coverage was significantly lower among those with no health-care visits in the past year (Table 2). Except for persons who had six to nine health-care visits and for persons who had no usual place for health care, influenza vaccination was significantly higher among persons with current asthma than it was for those without current asthma across all other characteristics, including number of health-care visits in the past 12 months, racial/ethnic group, having a usual place for health care, ability to pay for prescription drugs, and family income adjusted for family size (Table 2).

Among all persons, more than twice as many persons with health insurance coverage were vaccinated compared with those without health insurance coverage. Similarly, vaccination coverage was more than double among persons with a usual place for health care than among persons without a usual place for care (Table 2). Among persons with current asthma, 52.0% of those with a usual place for care were vaccinated, compared with 19.2% of those without a usual place for care (p<0.05). Regardless of asthma status, vaccination coverage was significantly lower among those who could not afford prescription drugs during the past 12 months than for those who could (Table 2).

Within the “all persons” and “without current asthma” groups, vaccination coverage for persons in families with incomes ≥250% the poverty threshold for family size was significantly higher than it was for persons in families with incomes less than the poverty threshold for family size. In addition, within the “all persons” and “without current asthma” groups, vaccination coverage was lower among non-Hispanic blacks and Hispanics than among non-Hispanic whites (Table 2). Among persons with current asthma, vaccination coverage was similar across racial/ethnic and income-to-poverty threshold ratio groups.

## Editorial Note

This report provides the first update of influenza vaccination coverage among the noninstitutionalized U.S. civilian population of persons with current asthma since ACIP recommended annual influenza vaccination for all persons aged ≥6 months beginning with the 2010–11 influenza season. Vaccination coverage among persons with current asthma has increased from 36% during the 2005–06 influenza season†† (3) to 50% during the 2010–11 season, with coverage increasing for all age groups.

What is already known on this topic?

Although persons with asthma historically have had higher influenza vaccination coverage than persons without asthma, coverage remains lower than *Healthy People 2020* targets.

What is added by this report?

Analysis of 2010 and 2011 National Health Interview Survey data shows that influenza vaccine coverage during the 2010–11 season among persons with asthma was 50%, up from 36% during the 2005–06 season, but coverage across all age groups remained well below *Healthy People 2020* targets of 80% for children aged 6 months–17 years and 90% for adults aged ≥18 years who are at high risk.

What are the implications for public health practice?

Measures that increase influenza vaccination among persons with asthma should be implemented. Interventions that have demonstrated benefits in similar settings include client reminders, reduced client out-of-pocket costs, and provider reminder systems.

This analysis supports findings from previous studies using NHIS data (3,6) indicating that having more health-care visits, health insurance coverage, a usual place for health care, and a higher family income relative to family size are significantly associated with higher vaccination coverage. Despite increased vaccination coverage among those with more health-care visits over the past year, more than half of persons with current asthma lacked current vaccination, suggesting that many health-care visits are missed opportunities for influenza-related education and vaccination.

ACIP has incrementally expanded the populations in the United States for whom seasonal influenza vaccination is recommended. Although children with asthma have been recommended to receive influenza vaccination annually, ACIP first recommended vaccination for all children aged 24–59 months regardless of risk status for the 2006–07 influenza season, and ACIP expanded that recommendation to include all children aged 5–18 years for the 2008–09 influenza season (7,8).§§ For the 2010–11 influenza season, ACIP recommended seasonal influenza vaccination for all persons aged ≥6 months (2). Although influenza vaccination coverage among persons with current asthma increased from 36.0% in 2005–06 to 49.6% in 2010–11, coverage among persons with current asthma increased the most among children aged 2–17 years (a 20.3 percentage point increase, from 32.5% to 52.8%). A similar increase was observed over the same period among children aged 2–17 years without current asthma (a 22.9 percentage point increase, from 15.9% to 38.8%). The increase suggests that the 2006 and 2008–2009 ACIP recommendations indicating vaccination of children regardless of risk status might have raised awareness about the importance of annual influenza vaccination among all children. Another possible contributing factor is that the 2009 H1N1 pandemic led to increased coverage during the 2010–11 influenza season.

The findings in this report are subject to at least five limitations. First, the limited sample size of persons with current asthma (n = 2,809) prevented reliable estimation of vaccination coverage of other sociodemographic subgroups not examined in this analysis. Second, because NHIS includes only those in the noninstitutionalized U.S. civilian population who agreed to participate, results might not be representative of other populations. Third, the NHIS response rates of 79.5% and 82.0% might have resulted in nonresponse bias, even after adjustment for nonresponse. Fourth, ACIP recommends that children aged 6 months–8 years who have never been vaccinated for influenza receive two vaccinations during the first influenza season to optimize immune response, but this analysis could not determine vaccination status from previous years (2). Finally, determination of asthma status and vaccination status in NHIS is made by self-report, which introduces the possibility of recall bias and misclassification (9).

These findings highlight the need to increase awareness of the importance of seasonal influenza vaccination for persons with asthma. The findings support recommendations made by the Task Force on Community Preventive Services, which recommends multicomponent interventions aimed at increasing influenza vaccination coverage (10). Specifically, the task force recommends the combination of one or more interventions to enhance access to vaccination services (e.g., reduced client out-of-pocket costs) with at least one provider-based or system-based intervention (e.g., provider reminder systems), and/or at least one intervention to increase client demand for vaccination (e.g., client reminders). In addition, to be consistent with ACIP recommendations, asthma education for health-care professionals could include recommendations for influenza vaccination for all patients with current asthma.

## Figures and Tables

**TABLE 1:** Influenza vaccination coverage* among persons aged ≥2 years, by current asthma status† and age group§ — National Health Interview Survey (NHIS), United States, 2010–11 influenza season

	All persons			Without current asthma			With current asthma			With asthma and attack in past 12 mos			With asthma and ED/urgent care visit in past 12 mos			With asthma and no attack or ED/urgent care visit in past 12 mos		
						
Age group (yrs)	No.¶	%	(95% CI)	No.	%	(95% CI)	No.	%	(95% CI)	No.	%	(95% CI)	No.	%	(95% CI)	No.	%	(95% CI)
2–17	6,900	40.2	(38.4–42.1)	6,186	38.8	(37.0–40.8)	714	52.8**	(47.3–58.6)	268	49.0	(40.9–57.7)	118	50.6	(37.8–65.0)	328	57.1	(49.0–65.5)
18–49	14,208	26.1††	(25.0–27.2)	13,118	25.4††	(24.3–26.5)	1,090	34.6**††	(30.7–38.8)	415	37.3††	(30.8–44.7)	118	35.2	(24.4–48.9)	557	32.4††	(26.9–38.8)
50–64	6,218	43.7††	(42.1–45.4)	5,652	42.0††	(40.4–43.7)	566	61.7**††	(55.8–67.7)	240	59.6	(50.0–69.3)	74	62.6	(46.7–78.5)	252	63.4	(55.1–71.7)
≥65	5,310	70.2††	(68.2–72.1)	4,871	69.7††	(67.7–71.7)	439	76.5**††	(70.2–82.2)	133	74.3††	(63.2–84.3)	50	83.7††	(68.9–94.1)	256	75.8††	(67.4–83.3)
**Total**	**32,636**	**38.5**	**(37.7–39.4)**	**29,827**	**37.5**	**(36.6–38.4)**	**2,809**	**49.6** **	**(47.0–52.3)**	**1,056**	**48.5**	**(44.3–52.8)**	**360**	**51.3**	**(44.3–58.7)**	**1,393**	**50.2**	**(46.3–54.2)**

**Abbreviations:** CI = confidence interval; ED = emergency department.

* Estimates are based on interviews conducted during September 2010–June 2011 and vaccination received during August 2010–May 2011. Estimates are based on responses by an adult to the following survey questions: “During the past 12 months, has [person] had a flu shot? A flu shot is usually given in the fall and protects against influenza for the flu season,” and ”During the past 12 months, has [person] had a flu vaccine sprayed in his/her nose by a doctor or other health professional? This vaccine is usually given in the fall and protects against influenza for the flu season,” and “During what month and year did you receive your most recent flu shot?” and “During what month and year did you receive your most recent flu nasal spray?” or responses by an adult about a child to the following questions: “During the past 12 months, has [child] had a flu vaccination? A flu vaccination is usually given in the fall and protects against influenza for the flu season,” and “During what month and year did [child] receive his/her most recent flu vaccine?”

† *Current asthma (child):* “Yes” response to the following survey questions, “Has a doctor or other health professional ever told you that [child] had asthma?” and “Does [child] still have asthma?” *Current asthma (adult):* “Yes” response to the following survey questions, “Have you ever been told by a doctor or other health professional that you had asthma?” and “Do you still have asthma?” *Without current asthma (child):* “No” response to one of the following survey questions: “Has a doctor or other health professional ever told you that [child] had asthma?” or “Does [child] still have asthma?” *Without current asthma (adult):* “No” response to one of the following survey questions: “Have you ever been told by a doctor or other health professional that you had asthma?” or “Do you still have asthma?” *Asthma attack or episode:* “Yes” response to the following survey questions, “During the past 12 months, have you [has child] had an episode of asthma or an asthma attack?” and “No” or “Don’t know/Refused” response to “During the past 12 months, have you [has child] had to visit an emergency room or urgent care center because of asthma?” *ED/urgent care visit*: “Yes” response to “During the past 12 months, have you [has child] had to visit an emergency room or urgent care center because of asthma?” and “Yes”, “No”, or “Don’t know/Refused” response to “During the past 12 months, have you [has child] had an episode of asthma or an asthma attack?” *No asthma attack or ED/urgent care visit:* “No” responses to the following survey questions: “During the past 12 months, have you [has child] had an episode of asthma or an asthma attack?” and “During the past 12 months, have you [has child] had to visit an emergency room or urgent care center because of asthma?” or “No” response to one of the two questions and “Don’t know/Refused” response to the other question.

§ Children were classified into age groups based on their age as of November 1, 2010. Adults were classified into age groups based on their age at time of NHIS interview.

¶ Unweighted sample size; percentages and CIs are weighted proportions.

** p<0.05 by t-test for comparisons between asthma groups (with current asthma versus without current asthma; with asthma and attack in past 12 months versus with asthma and no attack or ED/urgent care visit in past 12 months; and with asthma and ED/urgent care visit in past 12 months versus with asthma and no attack or ED/urgent care visit in past 12 months).

†† p<0.05 by t-test for comparisons between age groups, with persons aged 2–17 years as the reference group.

**TABLE 2:** Influenza vaccination coverage* among persons aged ≥2 years by current asthma status,† number of health-care visits,§ race/ethnicity,¶ health insurance coverage status,** usual place of care,†† inability to afford prescription drugs,§§ and income-to-poverty threshold ratio¶¶ — National Health Interview Survey (NHIS), United States, 2010–11 influenza season

	All persons			Without current asthma			With current asthma		
			
Characteristic	No.***	%†††	(95% CI)†††	No.***	%†††	(95% CI)†††	No.***	%†††	(95% CI)†††
**No. of health-care visits in past 12 mos**									
0§§§	5,804	14.7	(13.4–16.2)	5,576	14.5	(13.2–16.0)	228	19.7	(13.8–27.8)
1	5,713	29.8¶¶¶	(28.1–31.5)	5,407	29.3¶¶¶	(27.6–31.0)	306	39.3¶¶¶****	(31.5–48.3)
2–5	13,769	43.7¶¶¶	(42.5–45.0)	12,528	43.2¶¶¶	(41.9–44.5)	1,241	49.0¶¶¶****	(45.1–53.0)
6–9	3,211	51.4¶¶¶	(49.0–53.8)	2,836	51.3¶¶¶	(48.8–53.9)	375	52.1¶¶¶	(44.7–59.8)
≥10	4,023	55.9¶¶¶	(53.6–58.1)	3,377	54.2¶¶¶	(51.8–56.7)	646	65.7¶¶¶****	(60.1–71.3)
**Race/Ethnicity**									
White, non-Hispanic§§§	18,100	40.7	(39.5–41.8)	16,600	39.8	(38.6–41.0)	1,500	50.7****	(47.3–54.2)
Black, non-Hispanic	5,009	33.7¶¶¶	(31.5–36.0)	4,450	32.3¶¶¶	(29.9–34.9)	559	44.8****	(38.6–51.4)
Hispanic	6,700	32.8¶¶¶	(30.9–34.8)	6,174	31.5¶¶¶	(29.6–33.5)	526	49.3****	(42.4–56.7)
Other, non-Hispanic	2,827	39.3	(36.2–42.6)	2,603	38.2	(35.1–41.6)	224	51.0****	(41.2–61.7)
**Health insurance coverage**									
Covered§§§	26,794	42.7	(41.7–43.6)	24,390	41.6	(40.7–42.6)	2,404	53.6****	(50.9–56.4)
Not covered	5,738	17.8¶¶¶	(16.2–19.6)	5,343	17.2¶¶¶	(15.5–19.0)	395	25.8¶¶¶****	(19.9–33.1)
**Usual place for health care**									
Yes§§§	27,899	42.0	(41.0–42.9)	25,319	41.0	(40.0–42.0)	2,580	52.0****	(49.3–54.8)
No	4,366	15.5¶¶¶	(13.9–17.1)	4,154	15.3¶¶¶	(13.7–17.0)	212	19.2¶¶¶	(13.0–27.7)
**Could not afford prescription drugs in past 12 mos**									
Yes§§§	2,874	31.6	(29.1–34.2)	2,408	30.1	(27.3–33.0)	466	39.4****	(33.3–46.2)
No	29,748	39.2¶¶¶	(38.3–40.1)	27,407	38.1¶¶¶	(37.2–39.1)	2,341	51.5¶¶¶****	(48.7–54.4)
**Income-to-poverty threshold ratio**									
0–0.99§§§	6,326	33.1	(30.8–35.3)	5,644	31.3	(29.0–33.5)	682	46.9****	(40.6–53.3)
1.0–2.49	9,892	34.9	(33.3–36.6)	9,017	33.8	(32.1–35.6)	876	46.9****	(41.7–52.0)
2.5–4.49	8,230	38.4¶¶¶	(36.8–40.0)	7,565	37.3¶¶¶	(35.6–39.0)	665	50.3****	(44.7–56.0)
≥4.5	8,188	45.0¶¶¶	(43.3–46.6)	7,602	44.3¶¶¶	(42.6–45.9)	586	54.2****	(48.9–59.5)

**Abbreviation:** CI = confidence interval.

* Estimates are based on interviews conducted during September 2010–June 2011 and vaccination received during August 2010–May 2011. Estimates are based on responses by an adult to the following survey questions: “During the past 12 months, has [person] had a flu shot? A flu shot is usually given in the fall and protects against influenza for the flu season,” and ”During the past 12 months, has [person] had a flu vaccine sprayed in his/her nose by a doctor or other health professional? This vaccine is usually given in the fall and protects against influenza for the flu season,” and “During what month and year did you receive your most recent flu shot?” and “During what month and year did you receive your most recent flu nasal spray?” or responses by an adult about a child to the following questions: “During the past 12 months, has [child] had a flu vaccination? A flu vaccination is usually given in the fall and protects against influenza for the flu season,” and “During what month and year did [child] receive his/her most recent flu vaccine?”

† *Current asthma (child):* “Yes” response to the following survey questions, “Has a doctor or other health professional ever told you that [child] had asthma?” and “Does [child] still have asthma?” *Current asthma (adult):* “Yes” response to the following survey questions, “Have you ever been told by a doctor or other health professional that you had asthma?” and “Do you still have asthma?” *Without current asthma (child):* “No” response to one of the following survey questions: “Has a doctor or other health professional ever told you that [child] had asthma?” or “Does [child] still have asthma?” *Without current asthma (adult):* “No” response to one of the following survey questions: “Have you ever been told by a doctor or other health professional that you had asthma?” or “Do you still have asthma?”

§ Based on response to the question, “During the past 12 months, how many times have you seen a doctor or other health care professional about your own health at a doctor’s office, a clinic, or some other place? Do not include times you were hospitalized overnight, visits to hospital emergency rooms, home visits, dental visits, or telephone calls.”

¶ Based on responses to the following questions: “What race or races do/does [person] consider [yourself/herself/himself ] to be? Please select one or more of these categories,” and “Which one of these groups, that is [read groups selected] would you say best represents [person’s] race?”

** Health insurance coverage is at the time of the NHIS interview. Persons covered by Medicare, Medicaid, private insurance, Indian Health Service, military health care, state-sponsored health plans, or other government programs are considered covered. Persons not covered by any of these are considered not covered. This pertains to overall health insurance coverage and does not address whether vaccinations specifically are included in the coverage.

†† *Yes*: “Yes” or “There is more than one place” response to the question, “Is there a place that you usually go to when you are sick or need advice about your health?” *No*: “There is no place” response to the same question.

§§ *Yes*: “Yes” response to the question, “During the past 12 months, was there any time when you needed any of the following, but didn’t get it because you couldn’t afford it? Prescription medicines?” *No*: “No” response to the same question.

¶¶ Income-to-poverty threshold ratio is based on family income using the U.S. Census Bureau poverty thresholds for different family sizes. Family income was imputed when information was missing, using a multiple imputation methodology.

*** Unweighted sample size.

††† Percentages and CIs are weighted proportions.

§§§ Reference group used for pairwise significance testing within characteristic group and current asthma stratum.

¶¶¶ p<0.05 by t-test when compared with reference group within column.

**** p<0.05 by t-test for comparisons between “with current asthma” and “without current asthma” groups.

## Footnotes

* Additional information available at http://www.cdc.gov/flu/asthma/index.htm.

† From objective IID-12 (Increase the percentage of children and adults who are vaccinated annually against seasonal influenza). Available at http://www.healthypeople.gov/2020/topicsobjectives2020/objectiveslist.aspx?topicid=23.

§ *Current asthma (child):* “Yes” response to the following survey questions, “Has a doctor or other health professional ever told you that [child] had asthma?” and “Does [child] still have asthma?” *Current asthma (adult):* “Yes” response to the following survey questions, “Have you ever been told by a doctor or other health professional that you had asthma?” and “Do you still have asthma?”

¶ Children aged <2 years were not included for two reasons: 1) asthma diagnoses are considered unreliable in children at this age, and 2) there is a need for consistency with previous studies.

** Original estimates published in 2008 for the 2005–06 season were based on a different method for calculating season-specific influenza vaccination coverage, but those estimates were similar to estimates based on the Kaplan-Meier approach used in this report. Original and Kaplan-Meier estimates for persons with asthma by age group for the 2005–06 season were 36.2% versus 36.0% for persons aged ≥2 years, 29.3% versus 32.5% for children aged 2–17 years, 23.6% versus 22.3% for adults aged 18–49 years, 48.6% versus 45.9% for adults aged 50–64 years, and 75.7% versus 80.0% for adults ≥65 years, respectively. Original and Kaplan-Meier estimates were 14.3% versus 15.9% for children aged 2–17 years without asthma, respectively.

†† Additional information available at http://www.cdc.gov/flu/professionals/vaccination/coverage_1112estimates.htm#data.

§§ ACIP recommended adding children aged 5–18 years for annual influenza vaccination beginning in the 2008–09 influenza season, if feasible, but no later than the 2009–10 influenza season (8).
